# Vacancy Formation and Clustering Behavior in *δ*-MoN: A Systematic Density Functional Theory Study

**DOI:** 10.3390/nano15110810

**Published:** 2025-05-28

**Authors:** Jing Yu, Keda Wang

**Affiliations:** 1 College of Food and Pharmaceutical Engineering, Suihua University, Suihua 152061, China; yujinghrb@163.com; 2 Engineering Technology Center of Corn Processing and By-Products Biochemical Utilization, Suihua University, Suihua 152061, China

**Keywords:** vacancy, *δ*-MoN, bonding, electronic structure, density functional theory

## Abstract

Molybdenum nitrides are known to have a series of excellent physical properties owing to their unique bonding nature and electronic structure. However, the synthesized samples often exist in nonstoichiometric phases with structural defects (metal or non-metal vacancies), which may influence their performance. Based on the density functional theory, we theoretically studied the vacancy formation in *δ*-MoN. Various configurations that contained one single vacancy, divacancies, or trivacancies were constructed and systematically studied. It was found that Mo vacancy leads to significant electron loss at the vacant site while N vacancy results in excess electrons being trapped, forming a uniform electron gas region. Detailed analysis revealed that four types of binding clusters are encouraged to form in *δ*-MoN. The *V*_Mo_–*V*_N_ or *V*_N_–*V*_Mo_–*V*_N_ (with a sandwich structure) binding is owing to the positive and negative interaction between Mo and N vacancies. The *V*_N_–*V*_N_ or *V*_N_–*V*_N_–*V*_N_ binding is attributed to the overlap of electron density, but requires N vacancies to be distributed in a specific arrangement. Both Mo and N vacancies induce the anisotropic degradation of electronic conductivity in *δ*-MoN, with the extent of degradation governed by the vacancy type and concentration.

## 1. Introduction

Molybdenum nitrides, constituting a typical type of transition metal nitride, have attracted much attention due to their competitive properties such as superconductivity [1,2] and superhardness [3,4,5]. This class of materials can crystallize in different phases: *β*-Mo_2_N (tetragonal) [6,7], *γ*-Mo_2_N (cubic) [6,8], and *δ*-MoN (hexagonal) [6,9]. Among those phases, *δ*-MoN is of particular interest due to its special atomic arrangement and highly directional covalent bonds, which lead to a strong three-dimensional (3D) anion–cation bonding network, making it potentially able to achieve high hardness. *δ*-MoN has been considered the hardest superconducting metal nitride [10]. Its bulk modulus has been measured to be 345 GPa [4]. Meanwhile, the superconducting transition temperature (*T*_c_) of *δ*-MoN is up to 12–15 K [11,12], which is the second highest among the known metal nitrides, only slightly lower than the reported *T*_c_ of 16 K for *δ*-NbN [13]. This rare coexistence of superhardness and superconductivity makes *δ*-MoN particularly attractive for advanced technological applications such as wear-resistant superconducting coatings and electromechanical devices operating under extreme stress conditions.

Molybdenum nitrides are interstitial materials with nitrogen atoms occupying interstitial positions of metal atom arrangement. This type of compounds has an intrinsic nature in that it possesses homogeneity regions within which composition may deviate from stoichiometry [14]. Experimentally, multiple methods have been employed to synthesize *δ*-MoN, such as high–pressure and high–temperature synthesis [15,16], rf reactive magnetron sputtering [17,18], chemical vapor deposition [19], and the chemical solution method [20,21]. However, the synthesized samples often exist in nonstoichiometric phases, with the anion-to-cation ratio *x* either below or above unity (MoN*_x_ x* = 0.9–1.2) [17,19,22]. The relative deficiency (*x* < 1) or surplus (*x* > 1) of N atoms may result from the presence of unoccupied sites in non-metallic sublattice (non-metal vacancies) or the presence of unoccupied metallic sublattice sites (metal vacancies) [14,23]. It is known that the unique properties of metal nitrides are closely related to their bonding and electronic structures. High cohesion energy and high hardness are normally correlated with the covalent nature of bonding while superconductivity is related to the electronic structure and transport property. Structural imperfection may have a considerable effect on the properties of nonstoichiometric compounds as the existence of vacancies can fundamentally alter the bonding and electronic structures. For instance, even minor stoichiometric deviations may significantly alter the density of states near the Fermi level, thereby directly impacting superconducting transition temperatures and electronic transport properties. Thus, understanding the effects of vacancies on the bonding and electronic structures of such systems is essential for designing materials with desired properties. However, to the best of our knowledge, no relevant research has been devoted to nonstoichiometric *δ*-MoN.

As explained in this paper, we performed a systematic study of nonstoichiometric *δ*-MoN based on the density functional theory. Various configurations including one single vacancy, divacancies, or trivacancies were built and optimized to minimum energy structures. The vacancy formation energies, vacancy–vacancy interactions, and the effects of vacancies on the chemical bonding, electronic structures, and transport properties were theoretically studied. This paper is organized as follows. First, one neutral atom (Mo or N) was removed from the supercell to investigate the effects of one single vacancy on the structural, electronic, and magnetic properties of *δ*-MoN. Then, our calculations were extended to divacancies, followed by trivacancies, to identify multiple types of interactions between vacancies. Based on a detailed analysis of formation energy, binding energy, and the electron localization function (ELF), the origin of different vacancy–vacancy interactions and the possibility of forming vacancy clusters in *δ*-MoN were theoretically revealed. Finally, based on the analysis of the electronic structure, the influence of vacancies on the electronic transport properties of *δ*-MoN was systematically discussed. We hope our calculations can provide useful information on vacancy formation and its effects on the electronic properties of other nonstoichiometric metal nitrides.

## 2. Computational Details

Density functional theory (DFT) calculations were performed using the projector augmented wave (PAW) pseudopotentials as implemented in Vienna ab initio simulation package (VASP) [24]. As excessive vacancies may have a considerable effect on the phase stability, a 2 × 2 × 2 supercell containing 128 atoms was employed to simulate vacancy effects. By introducing 1–3 vacancies (corresponding to 1.56% to 4.69% vacancy concentration), we constrained the nitrogen stoichiometry to MoN*ₓ* with *x* = 0.95–1.05. This range was consistent with the nonstoichiometric compositions (*x* = 0.9–1.2) observed in synthesized *δ*-MoN samples under conventional conditions [17,19,22]. The exchange correlation functional was treated by Perdew–Burke–Ernzerhof form-generalized gradient approximation (GGA-PBE) [25]. Considering the strong correlated nature of transition metal *d* shell, we introduced the GGA+*U* method by a simplified version of approach of Dudarev et al. [26], where the effective Hubbard parameter was generally expressed as *U*_eff_ = *U* − *J*. *U* and *J* represented the energy cost of adding an extra electron at a particular site and the screened exchange energy, respectively. Here, an effective value of *U*_eff_ = 2 eV was selected for Mo-4*d* electrons, which was taken from the literature [27]. The plane-wave basis set with an energy cutoff of 500 eV was used for expanding the Kohn–Sham wave functions of valence electrons. The Brillouin zone integration was performed on a gamma-centered 3 × 3 × 3 *k*-point mesh. To ensure the convergence of calculated properties with respect to *k*-point sampling, we systematically evaluated the total energy for five systems (perfect supercell and four monovacancy configurations) using three *k*-point meshes: 2 × 2 × 2, 3 × 3 × 3, and 4 × 4 × 4. The variation in total energies as a function of *k*-point meshes is shown in Figure S1. It was found that the energy difference between 3 × 3 × 3 and 4 × 4 × 4 meshes was less than 0.001 eV·atom^−1^. Furthermore, Table S1 reveals that the formation energies calculated using 3 × 3 × 3 and 4 × 4 × 4 *k*-point meshes exhibited minor difference (≤0.01eV), confirming that the adopted 3 × 3 × 3 mesh provided fully converged results while maintaining computational efficiency. Both atomic positions and lattice parameters were fully relaxed until the forces on each atom were less than 0.01 eV·Å^−1^. All the electronic calculations were carried out based on optimized geometries.

## 3. Results and Discussion

**Figure 1:**
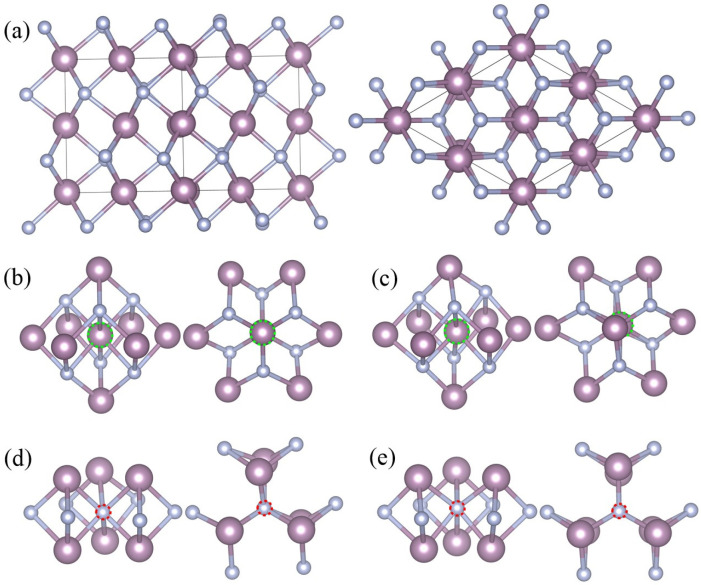
Crystal structure of *δ*-MoN (**a**) and local geometries of Mo_63_N_64_(2*a*) (**b**), Mo_63_N_64_(6*c*) (**c**), Mo_64_N_63_(2*b*) (**d**), and Mo_64_N_63_(6*c*) (**e**). Each structure contains a side view (**left**) and a top view (**right**). The pink spheres denote Mo atoms and white spheres denote N atoms. Mo and N vacancies are marked by green and red dashed circles, respectively.

The stable *δ*-MoN crystallizes in a distorted NiAs-type structure with a space group of *P*6_3_*mc* (186) [3,28], which is an interstitial compound with metal atoms forming a hexagonal lattice and non-metallic atoms located at the trigonal prismatic voids of the metallic lattice. Distinct alternating hexagonal layers of Mo and N atoms can be seen in the crystal structure of *δ*-MoN [c.f. Figure 1a]. In *δ*-MoN, there are two kinds of lattice points for Mo and N atoms. The Mo atoms are at the 2*a*(0, 0, *z*) (*z* = 0) and 6*c*(*x*, –*x*, *z*) (*x* = 0.4877, *z* = 0) sites while the N atoms are at the 2*b*(1/3, 2/3, *z*) (*z* = 0.2612) and 6*c*(*x*, –*x*, *z*) (*x* = 0.8302, *z* = 0.2438) sites [28]. Each unit cell of *δ*-MoN contains eight Mo atoms [two Mo(2*a*) atoms and six Mo(6*c*) atoms] and eight N atoms [two N(2*b*) atoms and six N(6*c*) atoms].

For the monovacancy configuration, a 2 × 2 × 2 supercell containing 64 Mo atoms and 64 N atoms was used to study the *δ*-MoN with one single vacancy. An isolated vacancy was created by removing one neutral atom (either Mo or N) from the supercell, which accounted for 1.56% vacancy concentration. Each time, one atom was removed from the supercell and a new monovacancy structure was built. There were four configurations considered in our calculations, namely, Mo_63_N_64_(2*a*) [one molybdenum vacancy at Mo(2*a*) site, *V*_Mo(2*a*)_], Mo_63_N_64_(6*c*) [one molybdenum vacancy at Mo(6*c*) site, *V*_Mo(6*c*)_], Mo_64_N_63_(2*b*) [one nitrogen vacancy at N(2*b*) site, *V*_N(2*b*)_], and Mo_64_N_63_(6*c*) [one nitrogen vacancy at N(6*c*) site, *V*_N(6*c*)_]. The corresponding local geometries are shown in Figure 1b–e.

We first performed a geometry optimization for the perfect supercell. It was found that the calculated lattice parameters (*a* = 11.581 Å, *c* = 11.305 Å) were in good agreement with experimental values (*a* = 11.483 Å, *c* = 11.237 Å) [28] with deviations within 1%, confirming the reliability of our computational approach. On the basis of equilibrium structure, four monovacancy configurations were constructed and relaxed to the minimum energy geometries and the obtained lattice parameters are listed in Table 1.

It is clearly seen in Table 1 that the presence of Mo or N vacancy had a nonnegligible effect on the geometry of *δ*-MoN, that is, the lattice parameters in the *a,b* directions were elongated whereas in the *c* direction, they were compressed. Four monovacancy configurations were all characterized by the shrinkage of the cell volume and a decrease in the *c*/*a* value. This case could be attributed to the atomic rearrangement caused by vacancies. Here, we take Mo_63_N_64_(6*c*) as an example to illustrate the reason for this change. In the hexagonal structure of *δ*-MoN, each Mo atom is surrounded by fourteen neighboring atoms. The first shell contains six first-nearest N atoms and the second shell involves eight second-nearest Mo atoms. The Mo-vacancy creation breaks adjacent Mo–N bonds and leads to a bipyramid-like hollow structure, as shown in Figure 1c, in which six Mo atoms in the *ab* plane make up a slightly deformed hexagon and other two Mo atoms along the *c* axis form two vertices of the bipyramid. The departure of Mo atom results in its neighboring atoms moving away from their original positions. Most evidently, two vertex Mo atoms of the bipyramid move inwards by a large magnitude of 0.24 Å, which is owing to the local collapse of the 3D anion–cation network caused by the missing Mo atom. Such a decrease in the Mo–Mo distance along the *c* axis is undoubtedly the origin of the shortening of lattice parameter *c*. Moreover, the significant change in the Mo–Mo distance breaks the force equilibrium between atoms and thus induces a certain degree of strain in the next bonds. To attain a new force equilibrium, neighboring atoms will follow the Mo displacements to reduce the tension. One consequence is that the hexagon Mo atoms of the bipyramid move outwards by about 0.02 Å, which is consistent with the slightly expanded supercell in the *a,b* directions. The Mo–N bond lengths around the Mo vacancy changed from the initial 2.09–2.26 Å to 2.11–2.26 Å in the study, with an average decrease of only 0.03 Å. Ultimately, the Mo vacancy at 6*c* position resulted in the shrinkage of the cell volume by 0.6% and the *c*/*a* value was reduced to 0.964. Similar changes can also be found in other monovacancy systems. However, it is noted that these changes in Mo_64_N_63_(2*b*) and Mo_64_N_63_(6*c*) are not as evident as those in Mo-monovacancy systems, which is due to the small radius of the N atom and its interstitial position. The remaining differences between Mo_63_N_64_(2*a*) and Mo_63_N_64_(6*c*) [or Mo_64_N_63_(2*b*) and Mo_64_N_63_(6*c*)] are attributed to the substantial differences in the atomic arrangements in these two configurations.

**Figure 2:**
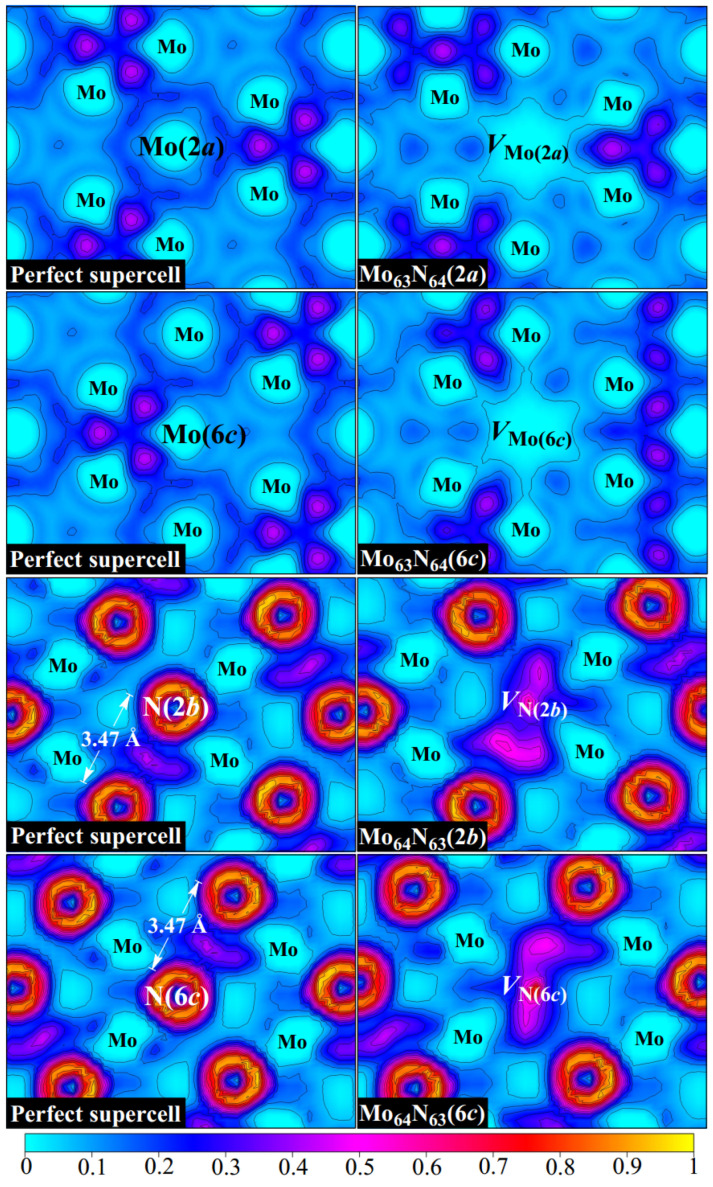
ELF distributions for the perfect and four monovacancy systems.

To further investigate the effects of vacancies on the bonding properties of *δ*-MoN, we analyze, in detail, the electron localization function (ELF) as introduced by Becke and Edgecombe [29,30,31]. This function describes the probability of finding two electrons with the same spin in space and provides a basis for a well-defined classification of chemical bonds [32]. The ELF takes the values ranging from 0 to 1, where 1 denotes the perfect localization of electrons, 0 represents low electron density, and 0.5 indicates a free electron gas. Figure 2 displays the ELF distributions of perfect *δ*-MoN as well as for four monovacancy systems.

**Figure 3:**
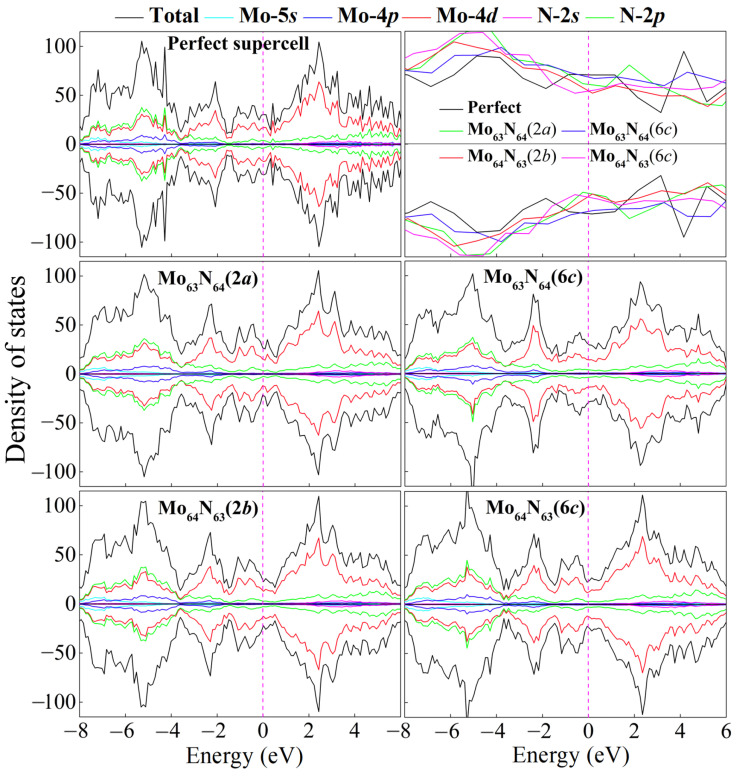
Total and partial density of states for the perfect and four monovacancy systems. Magnified DOS profiles focusing on the energy range of –0.8 to 0.6 eV are also given. The Fermi level is set as 0 eV.

We first examine the bonding characteristics of *δ*-MoN. The metallic bond, as a type of shared-electron interaction, possesses a distinctive feature wherein the ELF basins are big and extend over a region between bonded atoms. The ELF in the interaction region can be a non-nuclear maximum, like in covalent bonds, or a weak minimum as sparse electron gas [32]. It is clearly seen in Figure 2 that obvious metallic bonds exist in *δ*-MoN as the ELF basins (with the values of about 0.1–0.4) extend between metal atoms, which form continuous pathways for electrons to travel through. This is in line with the broad and delocalized *d* states throughout the valence and conduction bands, as is reflected from the density of states in Figure 3. The observed nonuniformity of electron localization between metal atoms is attributed to the uneven Mo–Mo distances determined by the crystal symmetry. It is noteworthy that due to the dispersed metallic bonds throughout the crystal, there is an enhanced localization of electrons between two neighboring N atoms that are far apart (the N–N distance is more than 3.4 Å, much larger than the sum of their covalent radii of 1.5 Å), which is favorable for stabilizing the structure of *δ*-MoN. Unlike the extended ELF basins of metallic bonds, covalent bonding is characterized by the presence of non-nuclear maxima on the line connecting two atoms. The ionic bonding is featured by a spherical ELF distribution around the atomic nucleus, which has no sharing electrons in the interstitial region. It is observed in Figure 2 that the ELF attains local maxima between Mo and N atoms. This is in accordance with the characteristics of covalent bonding. However, the electrons are not accumulated at the middle point of the Mo–N path but near the N site, showing the charge transfer to the N atom. According to the Bader charge analysis [33], each N atom in *δ*-MoN gains about 1.28 electrons, indicating a net shared charge of −1.72|e| with its adjacent Mo atoms, as the expected oxidation state is N^3−^. Therefore, the Mo–N interaction can be described as a partial covalent bond with some ionic component, which is consistent with the previous study [34]. In a word, a combined covalent–metallic–ionic bonding is found in the compound of *δ*-MoN.

Based on the structural analysis, we know that the formation of Mo or N vacancy breaks the force equilibrium between atoms and leads to atomic rearrangement. This rearrangement changes the interactions between atoms, thus redistributing the electron localization of *δ*-MoN, as can be seen in the ELF in Figure 2. As we know, the departure of the Mo atom will take away the electrons that were originally intended to be transferred to surrounding N atoms. Therefore, as the cost of Mo absence, the electron density around Mo vacancy tends to be depleted to compensate for the electron deficiency of N atoms, resulting in a large positive Mo hollow in *δ*-MoN. On the contrary, evident electron accumulation is observed at a N vacancy. The removal of the N atom breaks adjacent Mo–N bonds and releases electrons that should have been hybridized. Nevertheless, these released electrons are not completely returned to Mo atoms but are partially trapped at the N vacancy, forming a uniform electron gas region. Excess electrons make the N vacancy negatively charged and form a weak bonding with its surrounding atoms. Thus, we can say that the creation of Mo and N vacancies has different influences on the bonding of *δ*-MoN. The former results in significant electron deficiency in the Mo hollow while the latter leads to excess electrons being trapped at the N vacancy. Table 2 lists the oxidation states for six first-nearest-neighbor N (or Mo) atoms around *V*_Mo_ (or *V*_N_) in monovacancy systems. For the six first-nearest Mo atoms surrounding *V*_N_, the oxidation-state deviations from the perfect system are pronounced, with variations up to −0.27 [e.g., from +1.33 to +1.06 in Mo_64_N_63_(6*c*)], indicating significant local charge redistribution. In contrast, N atoms adjacent to *V*_Mo_ exhibit minimal charge perturbations, with differences not exceeding 0.10, suggesting weaker electronic coupling between N atoms and the vacant site. This difference in the charge response between Mo and N sublattices is likely driven by the higher electronegativity and smaller atomic radius of N, which limits its ability for charge redistribution. As compared to the perfect system, the charge redistribution around the vacancy presents broken-symmetry characteristics, which may induce local asymmetric polarization. However, the vacancy-induced perturbation decays rapidly with distance. Beyond the first coordination shell, the variation in oxidation states in outer Mo shells diminishes sharply, with a maximum deviation of 0.07, and eventually approaches zero at larger distances. Similarly, N atoms in outer shells show negligible changes (≤0.03), consistent with the short-range nature of vacancy effects. This rapid attenuation is mainly attributed to the delocalized electrons in *δ*-MoN, which can efficiently screen localized charge disturbances.

We further calculate the electronic density of states (DOS) for the perfect and four monovacancy structures, as shown in Figure 3. It can be seen that introducing one Mo or N vacancy into the superlattice changes the detailed structure of the DOS but does not fundamentally alter the metallic property of *δ*-MoN. Significant *p–d* hybridization between Mo-4*d* and N-2*p* orbitals (particularly in the deep bands of about –8 to –4 eV), as well as delocalized Mo-4*d* states dominating the Femi level, can also be observed in four monovacancy systems. However, due to the local damage of the transport channel caused by vacancies, a slight decrease in the DOS occurs at the Femi level, as is evidenced by the magnified DOS profiles focusing on the energy range of –0.8 to 0.6 eV in Figure 3. As we know, the density of states near the Fermi level plays an important role on the non-equilibrium transport properties of materials. A suppression of the DOS near the Fermi level inevitably reduces the population of delocalized electronic states participating in charge transport, thereby lowering the intrinsic carrier concentration. Meanwhile, vacancies act as scattering centers, disrupting the periodic potential of the lattice and enhancing electron–phonon or defect-mediated scattering events. This increases the scattering rate, further degrading the carrier mobility. The diminished carrier concentration coupled with degraded mobility will suppress the transport properties, which can be further confirmed by the calculated electronic conductivity in the final section.

**Figure 4:**
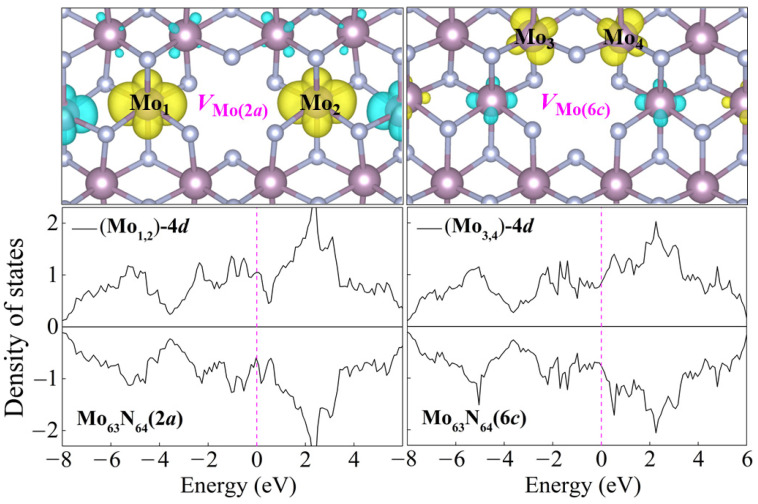
Spin charge density distributions and partial density of states of selected Mo atoms for Mo_63_N_64_(2*a*) and Mo_63_N_64_(6*c*). The Fermi level is set as 0 eV. Yellow and blue isosurfaces represent positive and negative spin densities, respectively.

Interestingly, a weak vacancy-induced magnetism is found in two Mo-vacancy-contained systems, Mo_63_N_64_(2*a*) and Mo_63_N_64_(6*c*). As is seen in the ELF in Figure 2, in contrast to the uniform electron gas present at the N vacancy, exposed dangling bonds pointing towards the Mo hollow obviously exist in Mo_63_N_64_(2*a*) and Mo_63_N_64_(6*c*). The removal of the Mo atom breaks surrounding Mo–Mo metallic bonds and creates dangling bonds around the vacancy, leaving Mo-4*d* electrons unpaired and induces magnetism in *δ*-MoN. To gain more insight into this Mo-vacancy-induced magnetism, the spin charge density distributions of Mo_63_N_64_(2*a*) and Mo_63_N_64_(6*c*) are presented in Figure 4. Clearly, the magnetic moments of these two systems mainly originate from the spin-polarized Mo-4*d* orbitals around the vacant site. The two main contributors, i.e., the two prominently spin-polarized Mo-4*d* orbitals [Mo_1,2_-4*d* around *V*_Mo(2*a*)_ or Mo_3,4_-4*d* around *V*_Mo(6*c*)_], provide magnetic moments of approximately 0.31 and 0.10 *μ*_B_ for Mo_63_N_64_(2*a*) and Mo_63_N_64_(6*c*), respectively. However, the Mo atoms adjacent to Mo_1,2_ or Mo_3,4_ are antiferromagnetically polarized due to the through-bond spin polarization. Ultimately, this antiferromagnetic Mo–Mo coupling results in 0.22 and 0.04 *μ*_B_ total magnetic moments for Mo_63_N_64_(2*a*) and Mo_63_N_64_(6*c*), respectively. It is not surprising that such small magnetic moments will not be apparently reflected from the total density of states (c.f. Figure 3). Thus, in each system, we select two Mo atoms (the two main magnetic contributors as mentioned above) to plot the partial density of states, as shown in Figure 4. The imbalance of spin-up and spin-down channels suggests the magnetic characteristics of Mo_63_N_64_(2*a*) and Mo_63_N_64_(6*c*).

The stability of a vacancy-contained system can be evaluated by the formation energy (*E*_f_), which is defined thus [35]:

(1) $$E_{f}=E_{\mathrm{tot}}-E_{\mathrm{per}}+\sum n_{i}\mu_{i}$$

Here, $E_{\mathrm{tot}}$ is the total energy of a vacancy-contained system and $E_{\mathrm{per}}$ is the energy of a perfect supercell. *n_i_* denotes the number of deficient atoms and $\mu_{i}$ denotes the chemical potential for species *i* (Mo or N). The chemical potentials of Mo and N ($\mu_{\mathrm{Mo}}$ and $\mu_{N}$) are not fixed but depend on growth conditions, ranging between Mo-rich (N-poor) and N-rich (Mo-poor) limits. The low and high values of $\mu_{Mo}$ correspond to N-rich and Mo-rich growth conditions, respectively, while $\mu_{N}$ exhibits the inverse dependence. To maintain structural stability, $\mu_{\mathrm{Mo}}$ and $\mu_{N}$ must satisfy the thermodynamic stability equilibrium: $\mu_{\mathrm{Mo}}+\mu_{N}=\mu_{\delta -\mathrm{MoN}}$, with the constraints $\mu_{N}$ ≤ $1/2\mu_{N_{2}}$ and $\mu_{\mathrm{Mo}}$ ≤ $\mu_{{\mathrm{Mo}}_{metal}}$. Under N-rich growth conditions, $\mu_{N}$ is estimated by the consideration of N_2_ molecule ($\mu_{N}=1/2\mu_{N_{2}}$), and $\mu_{Mo}$ is obtained from $\mu_{\mathrm{Mo}}=\mu_{\delta -\mathrm{MoN}}-\mu_{N}$. Under Mo-rich growth conditions, $\mu_{Mo}$ is determined as the energy of a molybdenum atom in bulk Mo and $\mu_{N}$ is evaluated by ${\mu_{N}=\mu }_{\delta -\mathrm{MoN}}-\mu_{\mathrm{Mo}}$. The calculated formation energies of four monovacancy systems are summarized in Table 1. It is known that formation energy is an important index that describes the energy cost for creating a vacancy in the host material. The smaller the formation energy is, the easier it is to create a vacancy. It is clearly seen in Table 1 that Mo-vacancy creation prefers N-rich conditions while N-vacancy creation depends more on Mo-rich conditions. Under equivalent growth conditions, Mo and N vacancies tend to be located at the 2*a* and 2*b* sites, respectively. The lowest vacancy formation energy is attained at the Mo(2*a*) lattice site under N-rich growth conditions, with a rather small value of 0.08 eV. A defective metallic sublattice is characteristic of nitrides that form in thin films and ultra-disperse powders [14]. The higher formation energy of Mo_63_N_64_(6*c*) as compared to that of Mo_63_N_64_(2*a*) can be understood by considering the local bonding around different Mo sites. Due to the mixed bonding nature of *δ*-MoN, each Mo atom that wants to leave has to break the surrounding Mo–N bonds and detach from the binding of Mo–Mo metallic bonds. The average Mo(6*c*)–N bond length is 2.18 Å, about 0.02 Å shorter than that of Mo(2*a*)–N. Moreover, the evident deformation of Mo(6*c*)-4*d* orbital is observed from the ELF in Figure 2, indicating a strong metal–metal interaction. These two factors suggest that more energy cost is required to remove a Mo atom from the 6*c* site, which means a higher formation energy. Similarly, stronger local bonding around the N(6*c*) site results in 0.34 eV energy higher than the vacancy formation at N(2*b*) site as the average Mo–N(6*c*) bond length is about 0.01 Å shorter than that of Mo–N(2*b*).

**Figure 5:**
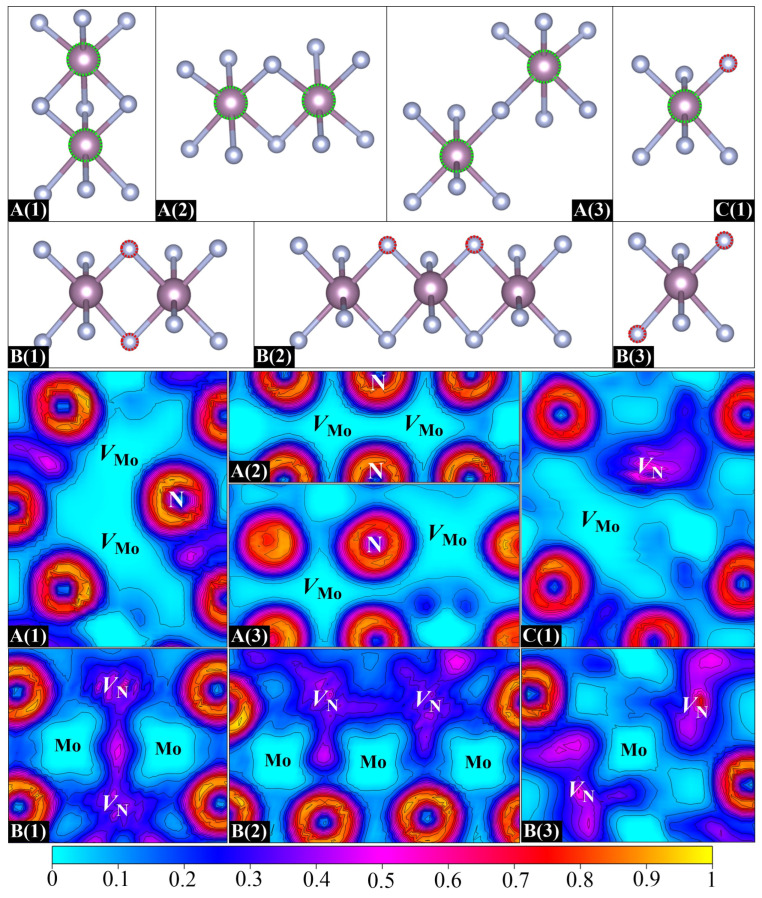
Seven designed divacancy configurations (**top**) and their corresponding ELF distributions on planes containing divacancy pairs and their adjacent atoms (**bottom**).

Next, we extend the analysis to a series of vacancy–vacancy pairs (divacancies) in *δ*-MoN. Each divacancy has been obtained by removing two neutral atoms from a 2 × 2 × 2 supercell, which amounts to 3.13% vacancy concentration. As there are two kinds of atoms (Mo and N) in *δ*-MoN, three types of vacancy–vacancy pairs have been considered in our calculations: Mo_62_N_64_ (containing two Mo vacancies, *V*_Mo_–*V*_Mo_ pair), Mo_64_N_62_ (containing two N vacancies, *V*_N_–*V*_N_ pair), and Mo_63_N_63_ (containing one Mo vacancy and one N vacancy, *V*_Mo_–*V*_N_ pair). This consideration generates seven different divacancies, as shown in Figure 5. These divacancy configurations are designed based on crystallographic symmetry and distinct vacancy pair arrangements within the superlattice framework. A(1), A(2), and A(3) are three *V*_Mo_–*V*_Mo_ pairs with three, two, and one bridging N atoms, respectively. In each plane, A(1), A(2), and A(3) exhibit distinct *X*:*Y* N-coordination ratios (2:2, 4:4, 4:2), where *X* and *Y* denote the first-shell nitrogen atoms bound to each vacancy in the *V*_Mo_–*V*_Mo_ pair. B(1), B(2), and B(3) are three *V*_N_–*V*_N_ pairs. B(1) has two bridging Mo atoms while B(2) and B(3) both contain a single bridging Mo atom but differ in lattice orientations. C(1) refers to a *V*_Mo_–*V*_N_ pair with two adjacent Mo and N vacancies. For all seven divacancy configurations, the separation between periodic images of vacancies ranges from 8.5–10.2 Å.

The interaction between vacancies in a cluster can be evaluated by the binding energy, which is defined thus [36]:

(2) $$E_{b}\left(V_{1}V_{2}\cdots V_{N}\right)=E_{f}\left(V_{1}\right)+E_{f}\left(V_{2}\right)+\cdots +E_{f}\left(V_{N}\right)-E_{f}\left(V_{1}V_{2}\cdots V_{N}\right),$$

Here, $E_{f}\left(V_{1}\right),$ $E_{f}\left(V_{2}\right),$ or $E_{f}\left(V_{N}\right)$ corresponds to the formation energy of a single vacancy and $E_{f}\left(V_{1}V_{2}\cdots V_{N}\right)$ denotes the formation energy of a vacancy cluster. The calculated formation and binding energies of seven different divacancies are listed in Table 3.

Here, we first focus on three *V*_Mo_–*V*_Mo_ pairs. Obviously, the binding energies of A(1), A(2), and A(3) are all negative, as is observed in Table 3. Moreover, the closer the *V*_Mo_–*V*_Mo_ distance is, the more negative the binding energy is. In particular, a binding energy of –1.14 eV is found for A(1), which corresponds to the nearest *V*_Mo_–*V*_Mo_ distance. Such a negative value suggests a strong *V*_Mo_–*V*_Mo_ repulsive interaction, which inevitably induces a higher formation energy than the creation of two isolated vacancies, indicating its thermodynamic instability. To better characterize this vacancy–vacancy interaction, the ELF distributions for different types of divacancies are displayed in Figure 5. As discussed above, the departure of the Mo atom removes its *ds* electrons and results in a significant lack of electrons around the Mo vacant site. As a result, once a *V*_Mo_–*V*_Mo_ pair is formed, the two electron-depleted neighbors may lead to a larger hollow area, as is evidenced by the ELF in Figure 5. The repulsion between two positively charged Mo vacancies is responsible for the negative binding energies of *V*_Mo_–*V*_Mo_ pairs. It is reasonable to suppose that Mo vacancies in real samples are often far enough apart to ensure that they do not interact with each other. However, different cases are found for *V*_N_–*V*_N_ pairs. Noticeably, a positive binding energy of 0.20 eV that demonstrates an attractive interaction is obtained for B(1). In contrast to the significant depletion of electrons of *V*_Mo_–*V*_Mo_ pairs, the electron density of B(1) is seen to be dispersed throughout two N vacancies, and particularly, there is a large portion accumulated in the interaction region, which suggests a tendency to form a *V*_N_–*V*_N_ binding cluster. It is noteworthy that this electron accumulation not only tightly binds the *V*_N_–*V*_N_ pair but also enhances the interaction between two shared Mo atoms. As can be seen in Figure 5, the local geometry of B(1) is characterized by a planar quadrilateral structure that is formed by two N vacancies and two shared Mo atoms. The ELF basins of both N vacancies extend to the middle of two shared Mo atoms, resulting in an increase in the electron density in the Mo–Mo interaction region. The Mo–Mo distance is correspondingly shortened by about 0.02 Å. A stable local quadrilateral structure is thus formed in B(1), which is beneficial for stabilizing the geometry. Unlike the geometry of B(1), two N vacancies in B(2) are located in two adjacent quadrilateral structures connected by one mediated Mo atom. This *V*_N_–*V*_N_ arrangement causes the electron density in each N vacancy to spread towards its respective quadrilateral center, which is featured by two relatively separated ELF basins with only few shared electrons in the *V*_N_–*V*_N_ interaction region. The small negative binding energy of –0.04 eV indicates that the *V*_N_–*V*_N_ repulsive interaction is slightly dominant in B(2). As for B(3), two well-separated ELF basins explain why it possesses a negative binding energy. Therefore, it can be concluded that, different from the distance dependence of *V*_Mo_–*V*_Mo_ interaction, the *V*_N_–*V*_N_ interaction relies more on the arrangement of two vacancies. With respect to the *V*_Mo_–*V*_N_ pair, an expected positive binding energy is found for C(1) as there is a positive and negative interaction between Mo and N vacancies, demonstrating the possible formation of *V*_Mo_–*V*_N_ binding clusters in *δ*-MoN.

**Figure 6:**
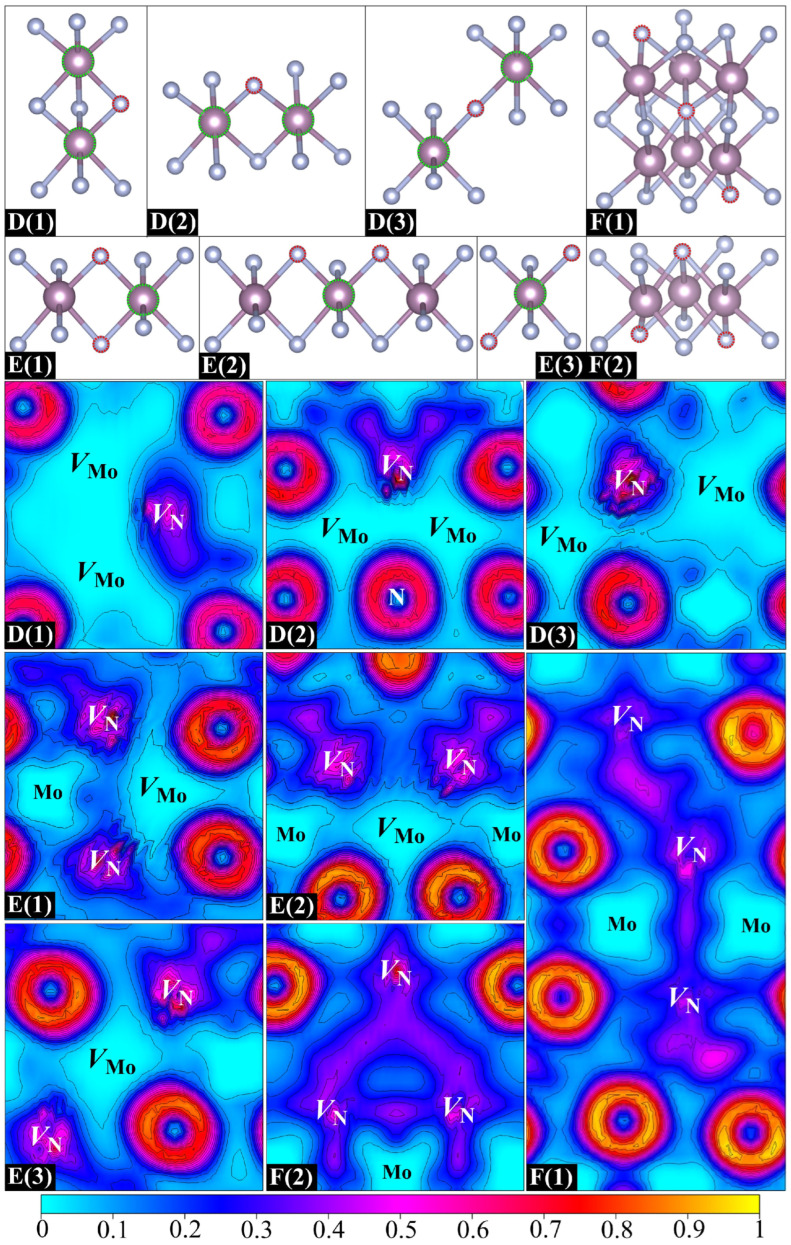
Eight designed trivacancy configurations (**top**) and their corresponding ELF distributions on planes containing trivacancy clusters and their adjacent atoms (**bottom**).

The above findings stimulate our interest in studying the possibility of forming larger vacancy clusters (trivacancies) in *δ*-MoN. The removal of three atoms from the supercell yields a vacancy concentration not exceeding 4.69%, which lies within the experimentally reported reference range [17,19,22]. Considering the *V*_Mo_–*V*_N_ attractive interaction and the arrangement dependence of *V*_N_–*V*_N_ pairs, three types of vacancy clusters were built: *V*_Mo_–*V*_N_–*V*_Mo_ (two Mo vacancies with one mediated N vacancy), *V*_N_–*V*_Mo_–*V*_N_ (two N vacancies with one mediated Mo vacancy), and *V*_N_–*V*_N_–*V*_N_ (three adjacent N vacancies). Figure 6 presents the eight built structures: D(1), D(2), and D(3) are three *V*_Mo_–*V*_N_–*V*_Mo_ clusters; E(1), E(2), and E(3) are three *V*_N_–*V*_Mo_–*V*_N_ clusters; and F(1) and F(2) are two *V*_N_–*V*_N_–*V*_N_ clusters. The *V*_Mo_–*V*_Mo_–*V*_Mo_ clusters are not discussed here due to the repulsion between adjacent Mo vacancies. For the majority of designed trivacancy configurations, the separation between periodic images of vacancies exceeds 8.5 Å. One exception is F(1), where the nearest distance between vacancy images is about 5.7 Å. To address potential finite-size effects, we have employed an enlarged 2 × 2 × 3 supercell to calculate the formation energy of F(1), yielding a value of 3.10 eV under N-deficient conditions, which is close to that obtained from the standard 2 × 2 × 2 supercell, with deviations limited to ~50 meV. Further analysis of the electronic transport property demonstrates the similarity between 2 × 2 × 2 and 2 × 2 × 3 supercells, confirming the validity of the smaller supercell for qualitative interpretation.

The formation and binding energies of eight trivacancies are listed in Table 4. It is clear that all three *V*_Mo_–*V*_N_–*V*_Mo_ clusters exhibit negative binding energies, especially for D(1). It is seen in the ELF distributions in Figure 6 that there is a significant electron loss in the N vacancy on the side adjacent to the Mo hollows. The remaining electrons are mainly distributed around the N vacant site rather than spreading into the mediating region between two Mo vacancies. Large positive Mo hollows can be obviously seen in the three *V*_Mo_–*V*_N_–*V*_Mo_ clusters, which is very similar to what is found in the case of *V*_Mo_–*V*_Mo_ pairs. Although the N vacancy has contributed some electrons to compensate for the electron deficiency of Mo vacancies, it is still far from fully mediating the direct *V*_Mo_–*V*_Mo_ interaction. The negative binding energy is proof that the direct *V*_Mo_–*V*_Mo_ repulsion is superior to the *V*_Mo_–*V*_N_ attraction in the *V*_Mo_–*V*_N_–*V*_Mo_ cluster. As for the *V*_N_–*V*_Mo_–*V*_N_ clusters of E(1) and E(2), their specific arrangements of vacancies result in two triangular structures, which can well balance the internal *V*_Mo_–*V*_N_ attraction and *V*_N_–*V*_N_ repulsion, thus leading to small negative binding energies. The *V*_N_–*V*_Mo_–*V*_N_ cluster of E(3) corresponds to a sandwich structure with one Mo vacancy in the interlayer and two N vacancies in the outer layers. This special structure ensures that the direct *V*_N_–*V*_N_ repulsion can be well shielded by the mediated Mo vacancy. The *V*_Mo_–*V*_N_ attraction thus becomes the dominant interaction in the cluster, giving rise to a positive binding energy. The above analysis of *V*_N_–*V*_N_ pairs offers us some clues for designing *V*_N_–*V*_N_–*V*_N_ clusters. F(1) and F(2) are two designed *V*_N_–*V*_N_–*V*_N_ clusters, both composed of two connected planar quadrilateral structures. Their *V*_N_–*V*_N_–*V*_N_ angles are about 150 and 50 degrees, respectively. It is evident from Figure 6 that the electron densities of three N vacancies in F(1) are well connected and form a uniform electron gas passing through the entire vacancy region, indicating the formation of a *V*_N_–*V*_N_–*V*_N_ binding cluster, which is consistent with its positive binding energy. Nevertheless, unlike the extended ELF basin in F(1), a triangular electron gas region is formed in F(2) owing to its smaller *V*_N_–*V*_N_–*V*_N_ angle. The structure of F(2) can be regarded as a folded F(1), with a significant decrease in the *V*_N_–*V*_N_–*V*_N_ angle by about 100 degrees. The distance between two endpoint N vacancies in F(2) is dramatically reduced as compared to that of F(1), thus leading to a B(2)-type *V*_N_–*V*_N_ repulsive interaction. The structural stability of F(2) is accordingly disrupted, resulting in a smaller binding energy than that of F(1). Therefore, it can be said that the N vacancies in *δ*-MoN prefer to extend along the diagonal of interconnected quadrilaterals in adjacent N layers rather than clustering in the local structure.

**Figure 7:**
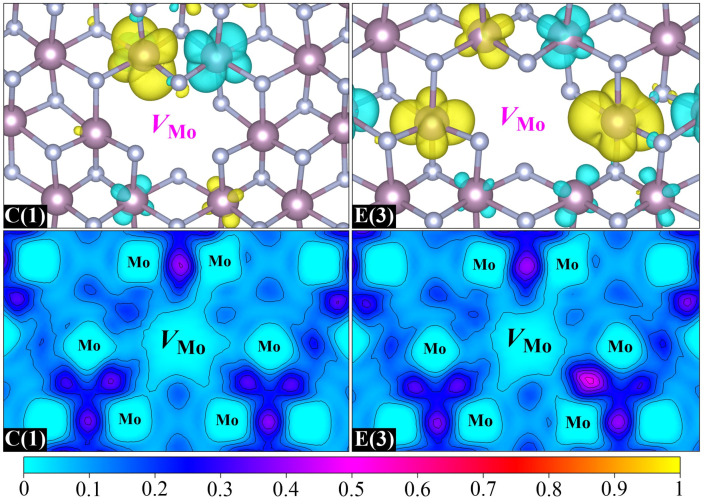
Spin charge density isosurfaces (**top**) and ELF distributions (**bottom**) with Mo vacancies located at central sites. The left and right pairs correspond to two distinct Mo-vacancy-contained configurations, divacancy C(1) and trivacancy E(3), respectively.

Then, we investigate the magnetic properties of two stable Mo-vacancy-contained binding clusters, namely, C(1) and E(3). Their spin charge density and ELF distributions are displayed in Figure 7. It is clear that the magnetic moments of C(1) and E(3) mainly come from the spin polarization of Mo-4*d* orbitals around the Mo vacancy, which is similar to the case of Mo-monovacancy systems. It is noteworthy that the maximum magnetic moment contributed by a single *d* orbital in C(1) is as high as 0.27 *μ*_B_, and increases to 0.30 *μ*_B_ in E(3), which is almost equivalent to the magnetic moment offered by the two main contributors in Mo_63_N_64_(2*a*) (0.31 *μ*_B_). This indicates that the formation of the *V*_Mo_–*V*_N_ or *V*_N_–*V*_Mo_–*V*_N_ binding cluster is beneficial for enhancing the Mo-vacancy-induced magnetism, which can be further confirmed by the ELF distributions. Once the *V*_Mo_–*V*_N_ or *V*_N_–*V*_Mo_–*V*_N_ binding is formed in *δ*-MoN, the significant electron loss caused by the departure of the Mo atom could be compensated for by the excess electrons in the N vacancy. As a result, more unpaired electrons will be left around the Mo vacant site, which is the origin of larger magnetic moments. The antiferromagnetic Mo–Mo interaction is also nonnegligible in the two binding clusters, which results in 0.13 and 0.29 *μ*_B_ total magnetic moments for C(1) and E(3), respectively. Similar to Mo-monovacancy systems, the total density of states for C(1) and E(3) exhibits weak spin polarization (Figure S2), indicating the spatially localized nature of spin polarization around the vacancy site.

Finally, we calculate the electronic conductivities at the temperature of 300 K for the perfect supercell and six selected stable vacancy-contained systems based on the semi-classical Boltzmann theory [37]. In this framework, the electronic conductivity is defined in units’ relaxation time. The calculated results are summarized in Table 5. The conductivities along all three crystallographic axes (*XX*: *a*-axis, *YY*: *b*-axis, ZZ: *c*-axis) have been evaluated, with average values provided for comparative analysis.

It is evident from Table 5 that the perfect supercell exhibits strong intrinsic anisotropy, with *YY*-direction conductivity (4.072 × 10^19^ S/m·s) exceeding those of the *XX* and *ZZ* directions by a factor of ~3.1, which originates from anisotropic electronic delocalization governed by crystallographic symmetry. The introduction of vacancies universally suppresses conductivity due to the dual effects of reduced carrier density near the Fermi level (c.f. Figure 3 and Figure S2) and degraded mobility. However, the extent of degradation and its anisotropy depend on the vacancy type and concentration. For instance, a single Mo vacancy [Mo_63_N_64_(2*a*)] preferentially disrupts *YY*-direction transport, reducing by 34.6%, suggesting Mo-dominated conduction channels along *b*-axis, while a single N vacancy [Mo_64_N_63_(2*b*)] severely compresses *ZZ*-direction conductivity, lowering by 41.2%, highlighting nitrogen’s critical role in facilitating interlayer charge transfer along the *c*-axis. Multi-vacancy configurations amplify these effects significantly. Taking F(1) as an example, triple N vacancies induce an 81.6% reduction in *ZZ*-direction conductivity, as compared to that of the perfect supercell. Calculations performed on a larger 2 × 2 × 3 supercell with triple N vacancies results in a *ZZ*-direction conductivity of 0.229 × 10^19^ S/m·s and an average conductivity of 0.988 × 10^19^ S/m·s, suggesting the disruption of transport channels along the *c*-axis, consistent with the behavior observed in the 2 × 2 × 2 supercell. Noticeably, Mo vacancies introduce weak spin asymmetry between spin-up and spin-down conductivities, consistent with the emergence of local magnetism in Mo-vacancy-contained systems, while N vacancies exhibit negligible spin polarization. The anisotropic conductivity degradation aligns with the directional disruption of transport pathways. The *YY* direction’s sensitivity to Mo vacancies implies the dominance of axial Mo–Mo bonding networks in *b*-axis conduction. The ZZ direction’s sensitivity to N vacancies suggests their critical function in mediating vertical charge transport across layers.

In summary, this study presented a detailed theoretical analysis on the vacancy formation and clustering behavior in *δ*-MoN, revealing the effects of vacancies on the bonding, electronic structure, magnetism, and transport properties. Nevertheless, the influence of excess vacancies on other important properties, such as phase stability and mechanical performance, remains underexplored. This highlights the necessity for future investigations and provides guidance for our subsequent work.

## 4. Conclusions

Based on the density functional theory, we performed a systematic investigation of the vacancy formation and vacancy-clustering behavior in *δ*-MoN. The effects of vacancies on the bonding, electronic structure, magnetism, and transport properties of *δ*-MoN were theoretically studied. Our calculations indicate that the formation of vacancies in *δ*-MoN relies not only on growth conditions but also on the vacancy positions. The Mo vacancy located at the 2*a* site is found to have the lowest formation energy under N-rich growth conditions. The presence of vacancies leads to atomic rearrangement and thereby changes the electron distributions. The Mo vacancy results in significant electron loss at the vacant site while the N vacancy leads to excess electrons being trapped, forming a uniform electron gas region. An interesting vacancy-induced magnetism can be observed in Mo-vacancy-contained systems as the departure of the Mo atom creates metallic dangling bonds around the vacancy and leaves Mo-4*d* electrons unpaired. The calculated binding energies together with the ELF distributions indicate that four types of binding clusters are encouraged to form in *δ*-MoN. The *V*_Mo_–*V*_N_ binding is supported by the positive and negative interaction between Mo and N vacancies. The *V*_N_–*V*_Mo_–*V*_N_ binding can only be achieved in a sandwich structure, where the direct *V*_N_–*V*_N_ repulsion can be well shielded by the mediated Mo vacancy. The *V*_N_–*V*_N_ (or *V*_N_–*V*_N_–*V*_N_) binding can be realized when two N vacancies are in the same quadrilateral (or there are three N vacancies in two N-vacancy-connected quadrilaterals with an extended *V*_N_–*V*_N_–*V*_N_ angle) as this vacancy arrangement can facilitate electron density overlap and avoid repulsion. The formation of *V*_Mo_–*V*_N_ or *V*_N_–*V*_Mo_–*V*_N_ binding clusters is beneficial for enhancing Mo-vacancy-induced magnetism as more unpaired electrons are left around the Mo hollow due to the electron compensation by the N vacancy. Both Mo and N vacancies induce the anisotropic degradation of electronic conductivity in *δ*-MoN. Mo vacancies preferentially disrupt in-plane charge transport via symmetry-breaking perturbations to Mo–Mo bonding networks, manifesting as spin-polarized current asymmetry, whereas N vacancies predominantly suppress interlayer conduction by destabilizing nitrogen-mediated charge-transfer pathways.

## Figures and Tables

**Table 1:** Calculated lattice parameters for both of perfect and monovacancy configurations and the corresponding formation energies (*E*_f_).

Configurations	*a*,*b* (Å)	*c* (Å)	*c*/*a*	Volume (Å^3^)	*E*_f_ (eV)	
					Mo-Rich	N-Rich
Perfect supercell	11.581	11.305	0.976	1312.9		
Mo_63_N_64_(2*a*)	11.644	11.215	0.963	1308.4	0.84	0.08
Mo_63_N_64_(6*c*)	11.623	11.207	0.964	1305.6	1.18	0.42
Mo_64_N_63_(2*b*)	11.650	11.237	0.965	1311.1	0.65	1.41
Mo_64_N_63_(6*c*)	11.615	11.262	0.970	1311.8	0.99	1.75

**Table 2:** Calculated oxidation states for six first-nearest-neighbor N (or Mo) atoms around *V*_Mo_ (or *V*_N_) in monovacancy systems. The corresponding oxidation states of the perfect supercell are also given for comparison.

Six First-Nearest N Atoms Around *V*_Mo_				Six First-Nearest Mo Atoms Around *V*_N_			
Mo_64_N_64_	Mo_63_N_64_(2*a*)	Mo_64_N_64_	Mo_63_N_64_(6*c*)	Mo_64_N_64_	Mo_64_N_63_(2*b*)	Mo_64_N_64_	Mo_64_N_63_(6*c*)
−1.28	−1.21	−1.27	−1.26	+1.25	+1.02	+1.32	+1.10
−1.28	−1.21	−1.28	−1.19	+1.27	+1.06	+1.25	+1.05
−1.27	−1.19	−1.28	−1.19	+1.26	+1.06	+1.26	+1.10
−1.28	−1.21	−1.28	−1.21	+1.25	+1.09	+1.33	+1.06
−1.27	−1.24	−1.28	−1.21	+1.27	+1.08	+1.25	+1.07
−1.28	−1.21	−1.27	−1.17	+1.26	+1.09	+1.26	+1.06

**Table 3:** The vacancy–vacancy distance (*d*) and formation (*E*_f_) and binding energies (*E*_b_) of various divacancies.

Divacancies	*d* (Å)	*E*_f_ (eV)		*E*_b_ (eV)
		Mo-Rich	N-Rich	
A(1)	2.83	2.82	1.30	–1.14
A(2)	2.90	2.42	0.90	–0.67
A(3)	4.12	2.15	0.63	–0.40
B(1)	3.28	1.93	3.45	0.20
B(2)	2.87	2.16	3.69	–0.04
B(3)	4.35	2.17	3.69	–0.19
C(1)	2.09	1.79	1.79	0.19

**Table 4:** Calculated formation (*E*_f_) and binding energies (*E*_b_) of trivacancies.

Divacancies	*E*_f_ (eV)		*E*_b_ (eV)
	Mo-Rich	N-Rich	
D(1)	3.72	2.95	–0.89
D(2)	3.22	2.45	–0.05
D(3)	3.00	2.23	–0.11
E(1)	3.04	3.80	–0.07
E(2)	2.98	3.74	–0.01
E(3)	2.97	3.73	0.15
F(1)	3.15	5.44	0.11
F(2)	3.25	5.54	0.01

**Table 5:** Calculated electronic conductivity (×10^19^ S/m·s) of different configurations at 300 K.

Configurations	Vacancy Type	*XX*	*YY*	*ZZ*	Average	Spin-Up	Spin-Down
Perfect supercell		1.317	4.072	1.324	2.238	1.120	1.118
Mo_63_N_64_(2*a*)	*V*_Mo_	1.450	2.665	1.049	1.721	0.851	0.870
Mo_64_N_63_(2*b*)	*V*_N_	1.013	2.992	0.778	1.594	0.801	0.793
B(1)	*V*_N_–*V*_N_	1.745	2.001	0.439	1.395	0.697	0.698
C(1)	*V*_Mo_–*V*_N_	1.037	1.560	0.604	1.067	0.553	0.514
E(3)	*V*_N_–*V*_Mo_–*V*_N_	0.944	1.564	0.453	0.987	0.460	0.527
F(1)	*V*_N_–*V*_N_–*V*_N_	1.434	1.546	0.243	1.074	0.536	0.538

## Data Availability

The original contributions presented in this study are included in the article/Supplementary Materials. Further inquiries can be directed to the corresponding author.

## Supplementary Materials

The following supporting information can be downloaded at: https://www.mdpi.com/article/10.3390/nano15110810/s1, Figure S1: Dependence of total energies for different configurations on the *k*-point mesh; Table S1: Formation energies for the monovacancy systems correspond to different *k*-point meshes; Figure S2: Total and partial density of states for selected multiple-vacancy systems.
