# Supplementary material for: Vacancy Formation and Clustering Behavior in δ-MoN: A Systematic Density Functional Theory Study
Source: Nanomaterials (Basel). 2025 May 28;15(11):810. doi: 10.3390/nano15110810 (PMC12157999; doi:10.3390/nano15110810)
Supplement: Supplementary file 1 [file nanomaterials-15-00810-s001.zip › nanomaterials-3600585-supplementary.pdf]

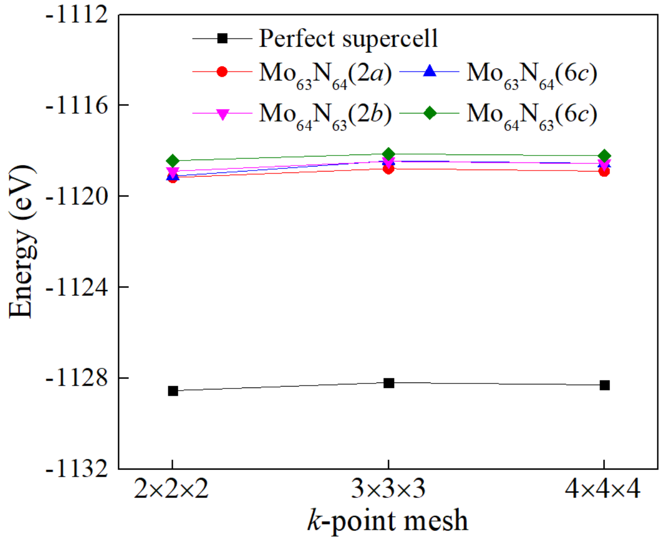

Figure S1. Dependence of total energies for different configurations on the *k*-point mesh.

Table S1. Formation energies for the monovacancy systems correspond to different *k*-point meshes.

Configurations	E_f (eV)					
	2×2×2		3×3×3		4×4×4	
	Mo-rich	N-rich	Mo-rich	N-rich	Mo-rich	N-rich
$Mo_{63}N_{64}$(2*a*)	0.82	0.06	0.84	0.08	0.85	0.09
$Mo_{63}N_{64}$(6*c*)	0.88	0.12	1.18	0.42	1.19	0.43
$Mo_{64}N_{63}$(2*b*)	0.57	1.33	0.65	1.41	0.65	1.41
$Mo_{64}N_{63}$(6*c*)	1.03	1.79	0.99	1.75	0.98	1.74

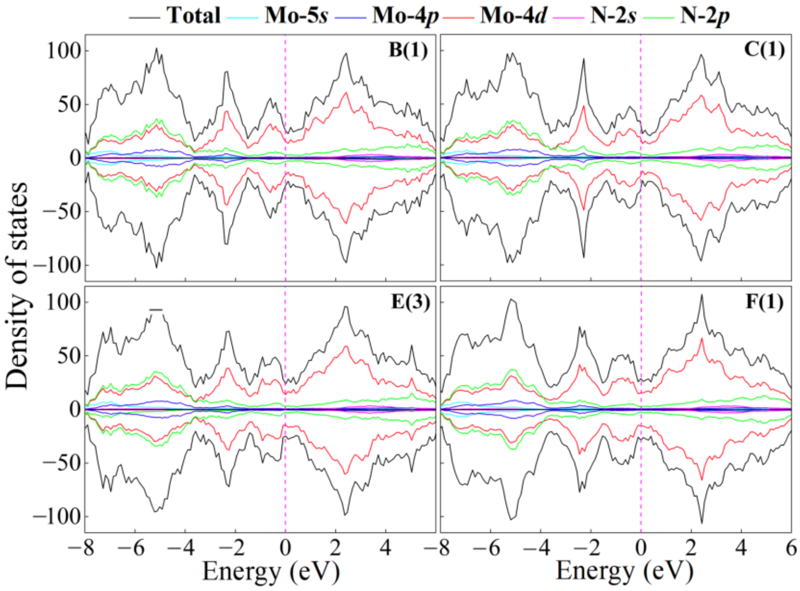

Figure S2. Total and partial density of states for selected multiple-vacancy systems.
